# Capsule robot pose and mechanism state detection in ultrasound using attention-based hierarchical deep learning

**DOI:** 10.1038/s41598-022-25572-w

**Published:** 2022-12-07

**Authors:** Xiaoyun Liu, Daniel Esser, Brandon Wagstaff, Anna Zavodni, Naomi Matsuura, Jonathan Kelly, Eric Diller

**Affiliations:** 1 Department of Mechanical and Industrial Engineering, University of Toronto, Toronto, ON M5S1A8 Canada; 2 Department of Mechanical Engineering, Vanderbilt University, Nashville, TN 37235 USA; 3 University of Toronto Institute of Aerospace Studies, University of Toronto, Toronto, ON M5S1A8 Canada; 4 Division of Cardiology, Department of Medicine, University of Toronto, Toronto, ON M5S1A8 Canada; 5 Department of Materials Science and Engineering and Institute of Biomedical Engineering, University of Toronto, Toronto, ON M5S1A8 Canada

**Keywords:** Biomedical engineering, Mechanical engineering

## Abstract

Ingestible robotic capsules with locomotion capabilities and on-board sampling mechanism have great potential for non-invasive diagnostic and interventional use in the gastrointestinal tract. Real-time tracking of capsule location and operational state is necessary for clinical application, yet remains a significant challenge. To this end, we propose an approach that can simultaneously determine the mechanism state and in-plane 2D pose of millimeter capsule robots in an anatomically representative environment using ultrasound imaging. Our work proposes an attention-based hierarchical deep learning approach and adapts the success of transfer learning towards solving the multi-task tracking problem with limited dataset. To train the neural networks, we generate a representative dataset of a robotic capsule within ex-vivo porcine stomachs. Experimental results show that the accuracy of capsule state classification is 97%, and the mean estimation errors for orientation and centroid position are 2.0 degrees and 0.24 mm (1.7% of the capsule’s body length) on the hold-out test set. Accurate detection of the capsule while manipulated by an external magnet in a porcine stomach and colon is also demonstrated. The results suggest our proposed method has the potential for advancing the wireless capsule-based technologies by providing accurate detection of capsule robots in clinical scenarios.

## Introduction

Wireless capsule robots have demonstrated great potential in tasks such as targeted drug delivery, biopsy sampling, and localized diagnostics of the gastrointestinal (GI) tract. Although endoscopic procedures are commonly conducted for diagnosis and treatment of the GI tract, endoscopy requires anesthesia and poses a risk of bowel rupture^1^. Minimally-invasive capsule-based technologies are promising alternatives to endoscopy. For procedures such as biopsy or targeted therapeutic delivery using wireless capsule robots, accurate and real-time tracking of the capsule location and operational status would be required.

Magnetic localization technology has been extensively investigated as an occlusion-free tracking scheme for pose estimation of wireless capsule endoscopy (WCE)^2^. A typical configuration is enclosing one or more small permanent magnets in the capsule, and external sensors are used to determine the pose of the capsule with the sensed magnetic field data. Existing methods achieve real-time precision with a high localization accuracy, but the capsule cannot be simultaneously actuated magnetically^3,4^ due to the undesirable interference with the localization system caused by the actuator. To address this problem, a coil system that is constrained in a three-by-three plane^5^ has been proposed to minimize the effect of the actuator on the sensor. Simultaneous magnetic actuation and localization of WCE has also been realized^6^, where the magnetic interference is eliminated by using an integral filter. However, such system is limited by the relatively low pose update frequency (about 0.5-1.0 Hz). Moreover, magnetic tracking does not allow for localization with respect to the GI-tract physiology, which would be needed for many applications such as targeted sampling, biopsy or therapeutic delivery. An US-guided capsule robot would address these barriers by offering tracking of the capsule using a safe, non-invasive imaging modality which also has the potential to localize the capsule with respect to GI tract location.

Compared with other medical imaging techniques such as magnetic resonance imaging (MRI) and X-ray, US imaging is favored for this application because it combines high temporal resolution and ionizing radiation-free imaging, at a lower cost and with easier access. Although US imaging is used in many clinical applications, there are challenges associated with imaging capsules using GI ultrasonography. The GI tract consists of 5 tissue layers in a tubular shape that are alternating echogenic and anechoic materials and will appear as bright and dark layers in an ultrasound B-mode scan. Furthermore, the interior of the GI tract is filled with a mixture of air, water, and digestive material, which manifests on an US B-mode image as the appearance of many speckles and contrasting light and dark areas^7^. In this environment, the accurate detection and tracking of capsule robots is very challenging.

Although US imaging is used manually as a diagnostic tool by skilled sonographers, automatic computer-based tracking of capsule robots is desired because (1) capsule robots with locomotion or mechanism actuation capability must be simultaneously tracked and controlled, which would present a major challenge to the operator since this requires the operator has a good knowledge of the dynamics and manipulation of both the capsule and US system; and (2) precise image interpretation by manually recognizing the device from background tissues and capsule on-board mechanism state in B-mode images is particularly difficult. An automated detection method would be used to aid a clinician in capsule operation, allowing for capsule tracking without the constant attention of a sonographer and providing more standardized feedback^8^. Such automated tracking would enable a fully closed-loop control along a path specified by a clinician through the anatomical workspace.

**Figure 1:**
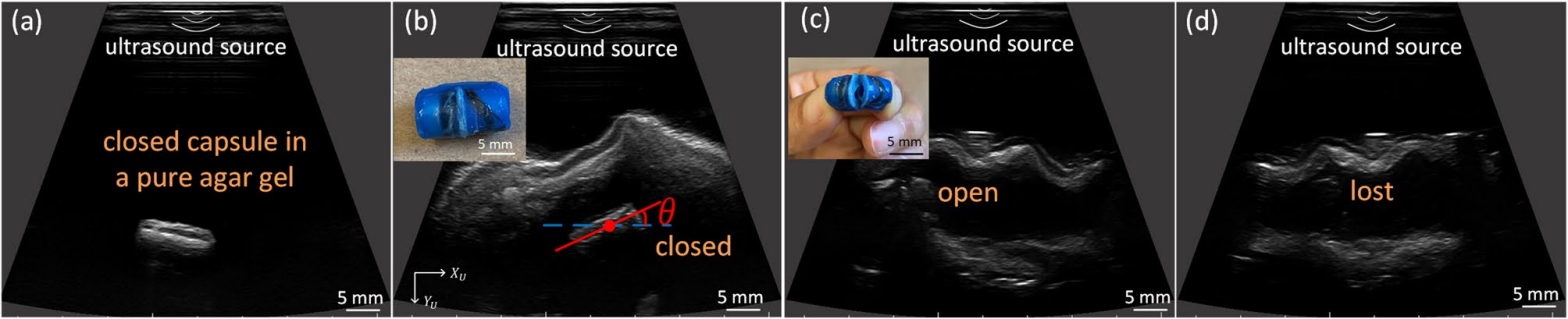
The capsule in a pure agar gel (**a**). In-plane 2D pose and the three mechanism states of the microcapsule including closed, open and lost-in-the- FOV in ultrasound images (**b**–**d**) acquired in an ex-vivo pig’s stomach. The centroid and orientation of the capsule are shown in (**b**) where the red line shows the orientation with respect to horizontal. The insets show the fabricated closed and open capsules.

In this work, we propose an attention-based hierarchical deep learning approach and adapt a well-trained CNN model on non-medical dataset for simultaneous 2D pose estimation and mechanism state detection of a milli-scale robotic capsule^9^ using ultrasound imaging in an ex-vivo porcine GI tract. The capsule consists of two permanent magnets encapsulated in a soft elastomer sample chamber. It has a magnetically-actuated sampling mechanism which causes the capsule to open up for sampling or therapeutic delivery when a magnetic field is applied. The diameter and length of the capsule are designed to be 8 mm and 14.5 mm, respectively. During clinical use, the capsule transits the GI tract following the oral administration and remains closed during locomotion, and is only activated to open for sampling or drug release when it arrives at a target location. Finally, the capsule can be retrieved via routine stool passage. We assume that the ultrasound probe is manually positioned to view the capsule robot, and only consider the motion of the robot agent while it is intersected by the ultrasound scanning plane. We consider three possible mechanism states of a capsule are closed, open, and lost-in-the-FOV (due to out-of-plane motion or occlusions induced by the surrounding tissues), as shown in Fig. 1b–d. In the lost-in-the-FOV case, the capsule is hard to visualize or only a small section with an irregular geometry is presented in an image. The 2D pose of a capsule in the ultrasound image coordinate system *U* is defined as $(x^{U}, y^{U}, \theta ^{U})$, the centroid position and orientation within the ultrasound imaging plane (Fig. 1b).

Our work seeks to address the challenging research issue on robust US-guided tracking of untethered capsule robots using a deep learning-based method with limited training data that can be employed in clinically representative environments. To this end, we explore the use of transfer learning to adapt a CNN model pre-trained by natural image dataset ImageNet^10^ to a different US dataset for the target multi-task tracking problem. The key contributions of the work include:

1. This work demonstrates simultaneous detection of pose and operational status of wireless capsule robots under US B-mode imaging in ex-vivo porcine GI tract. This allows for the integration into the conventional workflow of US-guided clinical procedures to assist clinicians for capsule use.
2. This work proposes a novel attention-based hierarchical deep learning approach for enhanced feature representation power, and is the first to adapt a CNN model pre-trained on a non-medical dataset to a clinical dataset for the target tracking tasks. The trained model developed on ex-vivo US images is of great clinical value and can serve as a pre-trained model for US-guided medical device tracking *in vivo*.
3. This work provides a large labeled dataset of US images in an ex-vivo clinically-relevant environment for benchmarking the development of tracking algorithms for micro-scale capsules.

## Related work

### Ultrasound imaging and tracking of micro/nanorobots

US-based tracking has previously been applied to various microrobots including a soft untethered gripper with a diameter of 2 mm to 5 mm^11^, a microswimmer that has a diameter and length of 100 $\upmu$m and 760 $\upmu$m^12^, a helical miniature robot^13^, and drug-coated microrobot in a vascular phantom^14^. US has also been used to localize collections of micro/nano-robots^15^. These studies use conventional computer vision-based methods, such as thresholding, blob detection, morphological filters and background subtraction that are capable of tracking targets in homogeneous and scarcely echogenic environments. Bourdeau et al.^16^ demonstrates the US imaging of even smaller microorganisms (100 $\upmu$m) with acoustic reporter genes (ARGs) that are based on gas vesicles and capable of enhancing US contrast significantly in the in-vivo GI tract of a mouse. However, objects in such small dimension without US contrast agents are difficult to be directly imaged using typical clinical US system. Moreover, Ren et al.^17^ presents a technique to detect metal surgical instruments in an ultrasound image based on the difference in ultrasonic properties between soft tissues and metal, which may not be robust for objects with low contrast to surrounding tissues. In addition to standard B-mode imaging, ultrasound Doppler imaging^18^ has also been applied to indirectly localizing collective cell microrobots in blood based on the induced Doppler signals from the surrounding red blood cells when the scanning plane does not intersect with the microrobots. In a recent work, ultrasound acoustic phase analysis (US-APA) based method^19^ has been proposed, in which the axial resolution of US imaging can be improved to 38 $\upmu$m by exploiting vibrations of the microrobot. This non-imaging based method enables the visualization of microrobots that are smaller than the US wavelength $\lambda$ (100-500 $\upmu$m for commercial system, operating in a range of 3-15 MHz) in highly echogenic biological tissue environments. However, it requires the access of radio frequency (RF) signal which is not commonly provided by clinical US systems.

### Deep learning-based methods

Computer vision algorithms that have been tested in simulated (phantom) environments are not representative of typical clinical use cases, in which contrast from the inhomogeneous tissue and surrounding air bubbles in the GI tract make in-vivo imaging significantly more challenging. This limitation motivates the use of deep learning-based approaches to accomplish improved tracking robotic capsules in the GI tract. The success of deep learning in various computer vision tasks^20,21^ offers the potential of applying those methods to the detection of interventional tools in biomedical images. Compared to conventional methods that are highly dependent on salient features and intensity gradients, convolutional neural networks (CNN) can automatically learn the task relevant feature representation from provided data in demanding imaging conditions.

There has been extensive research conducted on deep learning-based tracking of microrobots and interventional tools using different imaging modalities. Numerous works spatially localize the targets using RGB cameras and in surgical videos via deep learning-based semantic segmentation^22,23^. Others have used a region-based CNN that simultaneously provides localization and recognition of different surgical tools^24,25^. In addition to 2D localization, deep learning-based methods are proposed for 3D pose estimation of the endoscopic capsule robot^26^ and optical microrobots^27,28^ using a microscope-camera system and combined endoscopic camera and magnetic sensor data, respectively.

However, deep learning-based detection of medical devices in ultrasound images is still not fully explored and related studies are mostly limited to US-based needle and catheter tracking^29^. A multi-dimensional network is proposed for real-time catheter and needle detection in 3D ex-vivo porcine cardiac and chicken breast volumetric ultrasound images^30^. Similarly, a combined adaptive thresholding and Share-ConvNet method is proposed for catheter localization in 3D ex-vivo porcine cardiac US, and achieves an average localization error of 2.1 mm^31^. In addition, 2D biopsy needle detection is achieved in in-vivo kidney biopsy US images by using a tracking-by-segmentation method^32^. As a result, deep learning-based detection of untethered capsule robots using US feedback is still unexplored. Approaches in existing literatures can’t be applied to our work, since we are solving a different multi-task tracking problem. Moreover, unlike a needle that features an unique line-shape geometry and higher contrast due to the difference in ultrasonic properties between soft tissues and metal, the capsule doesn’t appear distinct compared with the surrounding tubular tissues in B-mode images. Thus, training a robust neural network model for accurate detection of capsule robots in such highly heterogeneous environment is more challenging.

### Transfer learning in medical image analysis

Deployment of transfer learning with state-of-the-art pre-trained CNN models has been emerging for analyzing medical images^33^ in recent years to address the lack of large labeled dataset in clinical practice for training a robust deep neural network exclusively from task-specific examples. Transfer learning has been proven promising for medical image analysis on various anatomical areas, among which Inception models pre-trained on non-medical ImageNet dataset are used as a feature extractor to analyze ultrasound images for the diagnosis of thyroid nodules in US images^34^ and staging liver fibrosis^35^. However, the implementation of transfer learning by adapting a CNN model pre-trained on non-medical dataset to US data for tracking capsule robots has never been reported, which is worthy of investigation.

**Figure 2:**
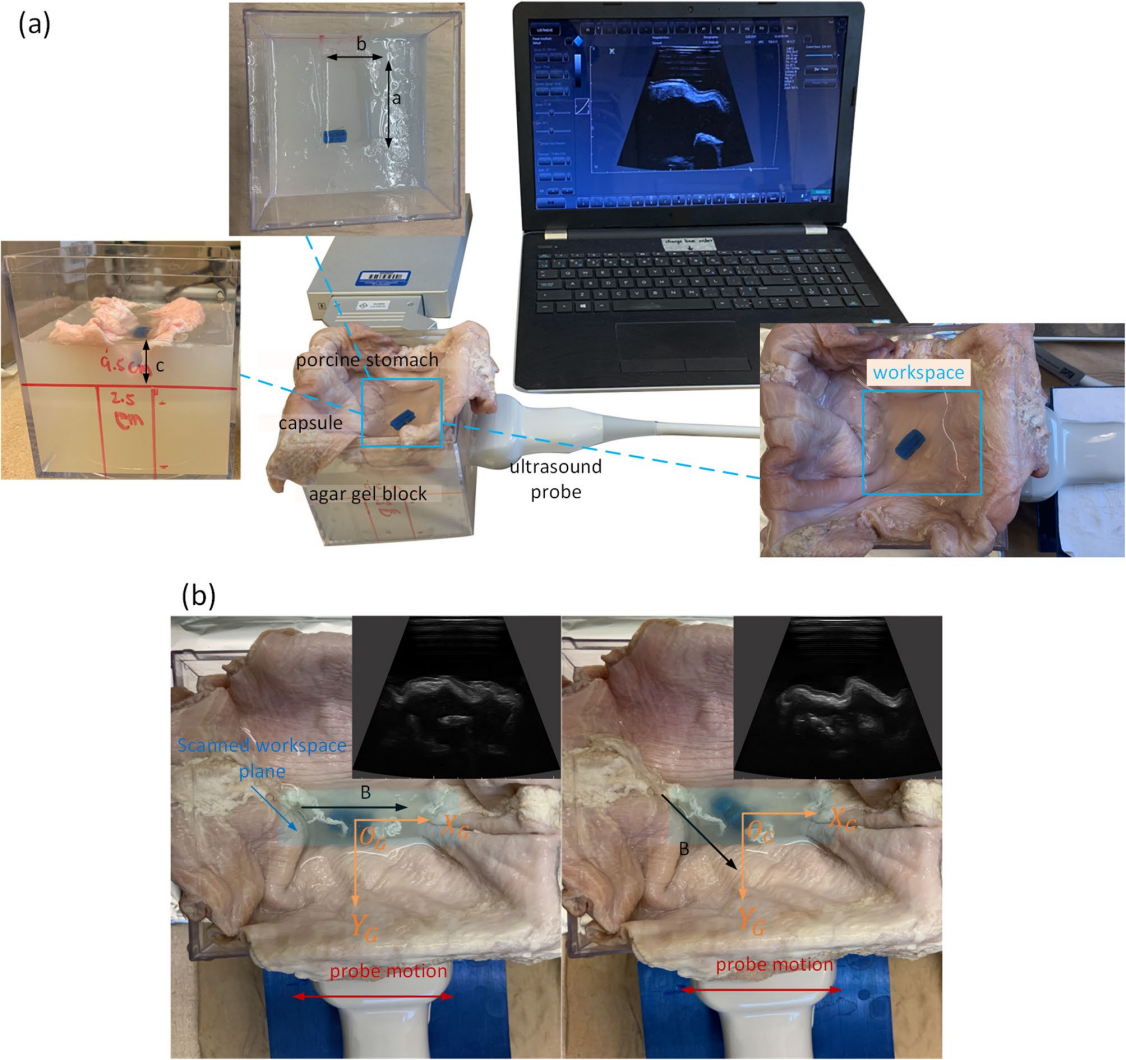
The experimental setup is depicted in (**a**), where the defined workspace is the embedded section of the stomach in the channel and the channel length $a = 1.4L$, the length of the probe, channel width $b = 3\,cm$ and depth $c\ge w$, the width of the probe. (**b**) shows the base dataset generation where the insets are the corresponding ultrasound images. *B* represents the external magnetic field and the red arrow indicates the translation of the probe during data collection.

## Results

### Experimental setup

The experimental setup is shown in Fig. 2, where the ultrasound probe is fixed to image the capsule from the side of the agar gel workspace, which contains a channel for the capsule to move in. Ultrasound gel is used to acoustically couple the probe to the imaging workspace to allow for ultrasound imaging through the container wall. An anatomically-representative imaging setup is constructed by placing a stomach tissue sample inside of the agar gel block. The use of ex-vivo stomach tissues can render a more representative imaging environment compared to the homogeneous agar or gelatin gel in existing literature. In order to replicate clinical GI ultrasonography, an anechoic non-absorbable polyethylene glycol (PEG) solution^36^ is used to fill the stomach. In addition, digestive enzymes powder (Webber Naturals) is dissolved in the solution to simulate the interior aqueous environment of the stomach. The mixed solution is used to fill the section of the stomach embedded in the channel, which defines a workspace for the capsule to move. The dimensions of the channel, including the length *a*, width *b*, and depth *c*, are designed to ensure that the capsule moves within the FOV of the probe.

### Performance evaluation

Given the ground truth centroid position in pixels and the orientation of the capsule in an ultrasound image $(x_{GTpixel,i}^{U}, y_{GTpixel,i}^{U}, \theta _{GT,i}^{U})$, where $i\in \{ 1,2,\ldots , N \}$ with *N* representing the total number of images in the test dataset. The pixel values are converted to metric coordinates (mm) by multiplying the pixel value with the scale factor of the probe. Then the mean position estimation error (PE) is calculated as

1 $$\begin{aligned} PE=\frac{1}{N}\sum _{i=1}^{N}\Vert P_{GT,i}^{U}-P_{i}^{U}\Vert \end{aligned}$$

where $P_{GT,i}^{U} = \sqrt{x_{GT,i}^{U2} + y_{GT,i}^{U2}}$ and $P_{i}^{U} = \sqrt{x_{i}^{U2} + y_{i}^{U2}}$ with $(x_{GT,i}^{U}, y_{GT,i}^{U})$ and $(x_{i}^{U}, y_{i}^{U})$ denoting the ground truth and estimated centroid position in millimeters. The mean orientation error (OE) is computed as $OE = \frac{1}{N}\sum _{i=1}^{N}\Vert \theta _{GT,i}^{U}-\theta _{i}^{U}\Vert$. Regarding state error, in this work we use state classification accuracy as the evaluation metric, which is computed as

2 $$\begin{aligned} Accuracy=\frac{N_{open}+N_{closed}+N_{lost}}{N} \end{aligned}$$

where $N_{open}$, $N_{closed}$, and $N_{lost}$ represent correct state detections for the three state classes.

### Hold-out test results

#### Mechanism state classification

**Figure 3:**
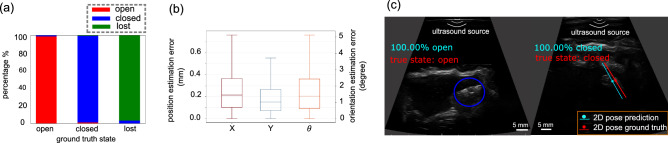
Hold-out test results for the mechanism state classification and 2D pose estimation. (**a**) shows the classification results for each status, (**b**) shows the error distributions of the 2D pose, and (**c**) are the sample test images for the status detection and 2D pose estimation.

We used a hold-out test set to evaluate the effectiveness of the training, which contains a total of 2839 images with 981, 969, and 889 image frames for the open, closed and lost capsules. The model achieves an accuracy of 97.83% with the classification results for each class and sample results shown in Fig. 3. Metrics for evaluating the neural network model are shown in Table 1. As shown in Fig. 3a, the trained model can classify each status correctly with high accuracy, while error cases occur mostly for classifying “closed” and “lost” status, as well as “closed” and “open” status. The model erroneously classifies some of the open capsules as closed because the open capsule looks similar to the closed one if the cavity only slightly opens or the surrounding tissues occlude the cavity. The model also has difficulty with classifying the lost and closed capsule in some cases by either recognizing the tissues as the closed capsule or vice versa due to the similarity between them.

**Table 1:** Metrics for evaluating the status classification on the hold-out test set.

Status	Precision	Recall	F1-score	Number of samples
Open	1.00	0.99	0.99	981
Closed	0.96	0.98	0.97	969
Lost	0.98	0.97	0.97	889

#### 2D pose estimation

The hold-out test set for the centroid position contains 2521 images of the closed capsule that is located at random positions and presents varying contrast in the images. The quantitative test results are shown in Fig. 3b, where the median errors are 0.21 mm and 0.15 mm for *x* and *y* coordinates, respectively. The mean error for the center point position is 0.24 mm, which corresponds to 1.7% of the capsule’s body length. The median error for the orientation is $1.3^{\circ }$ and the mean error is $2.0^{\circ }$ with the standard deviation of $4.1^{\circ }$. We consider a prediction with an error within $\pm 10^{\circ }$ to be accurate given the target application, and the metric of successful rate is defined as the percentage of the accurate orientation predictions, which is 97.8% for the hold-out test set. The sample test images in Fig. 3c show that the model can detect the capsule successfully despite the demanding imaging conditions, which demonstrate the generalization ability of the model on unseen test data.

**Figure 4:**
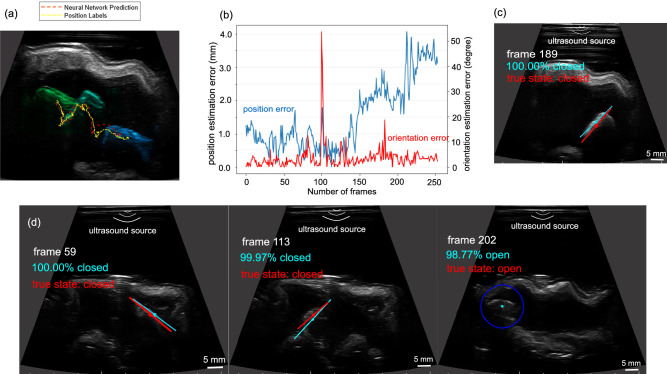
Ex-vivo pose estimation and mechanism state detection of the capsule. (**a**) shows the predicted versus the ground truth trajectories with a snapshot (**c**) from the video for the first trial, and (**b**) is the pose estimation error with respect to the frames in the first trial. (**d**) shows the snapshots from the video for the second trial.

### Ex-vivo pose estimation and mechanism state detection of the capsule robot

#### Ex-Vivo demonstration in a porcine stomach

To further demonstrate the neural network model capability, the model was used to detect the capsule in a different pig’s stomach. In the first trial, the capsule was actuated by a permanent magnet to move in a section of the stomach with a thinner tissue layer, where the capsule has good imaging contrast with a salient contour definition. The model detects the capsule in every frame precisely with estimation errors of 1.42 mm and 1.26 mm for *x* and *y* coordinates of the center point. The localization error is 1.76 mm, which corresponds to 12.1% of the body length. The ground truth and the predicted trajectories with the capsule in the initial, halfway, and final locations are plotted in Fig. 4a with the estimation errors in Fig. 4b. The successful rate of the orientation estimation is 96.9%, and the mean error is $3.5^{\circ }$, with a standard deviation of $4.7^{\circ }$. Angle ambiguity occurs at angles close to horizontal in a few frames, as shown in the supporting video. A sample test image is presented in Fig. 4c where the capsule is detected as “closed” with 100% accuracy through the entire locomotion sequence.

In trial 2, to demonstrate the potential of our proposed method for tracking both the 2D pose and operational status of the wireless capsule for biopsy sampling in the GI tract in real clinical applications, the capsule was moved from an initial location to the final location actuated by a permanent magnet on top of the workspace and kept closed during locomotion. Then the capsule was actuated by bringing the magnet closer to it for cavity opening and biopsy sampling. In this trial, the capsule was moved in a section of the middle portion of the stomach with a thicker tissue and internal fat layers. Here, the ultrasound waves are attenuated and backscattered significantly from the surrounding tissues, rendering an image with low contrast and lots of speckle. The model successfully detects the capsule in most frames where the capsule presents low visibility and irregular geometries (Fig. 4d) except for a few frames (Supporting Video). In addition, the model detects the mechanism closed and lost state of the capsule correctly during locomotion. However, when the capsule gradually opens or the tissue occludes the cavity, the neural network model has difficulty with recognizing the open state and erroneously detects the closed state (Supplementary Fig. S4a). As the cavity further opens, the model can detect the state as “open” correctly, and the model trained on the closed capsule can localize the open capsule in the image (Fig. 4d). As shown in the supporting video, in this trial, the path is not smooth, and the capsule moves back and forth with some abrupt motions because the capsule was stuck and entangled by the internal thick tissue layer in the middle portion of the stomach.

#### Ex-vivo demonstration in a porcine colon

**Figure 5:**
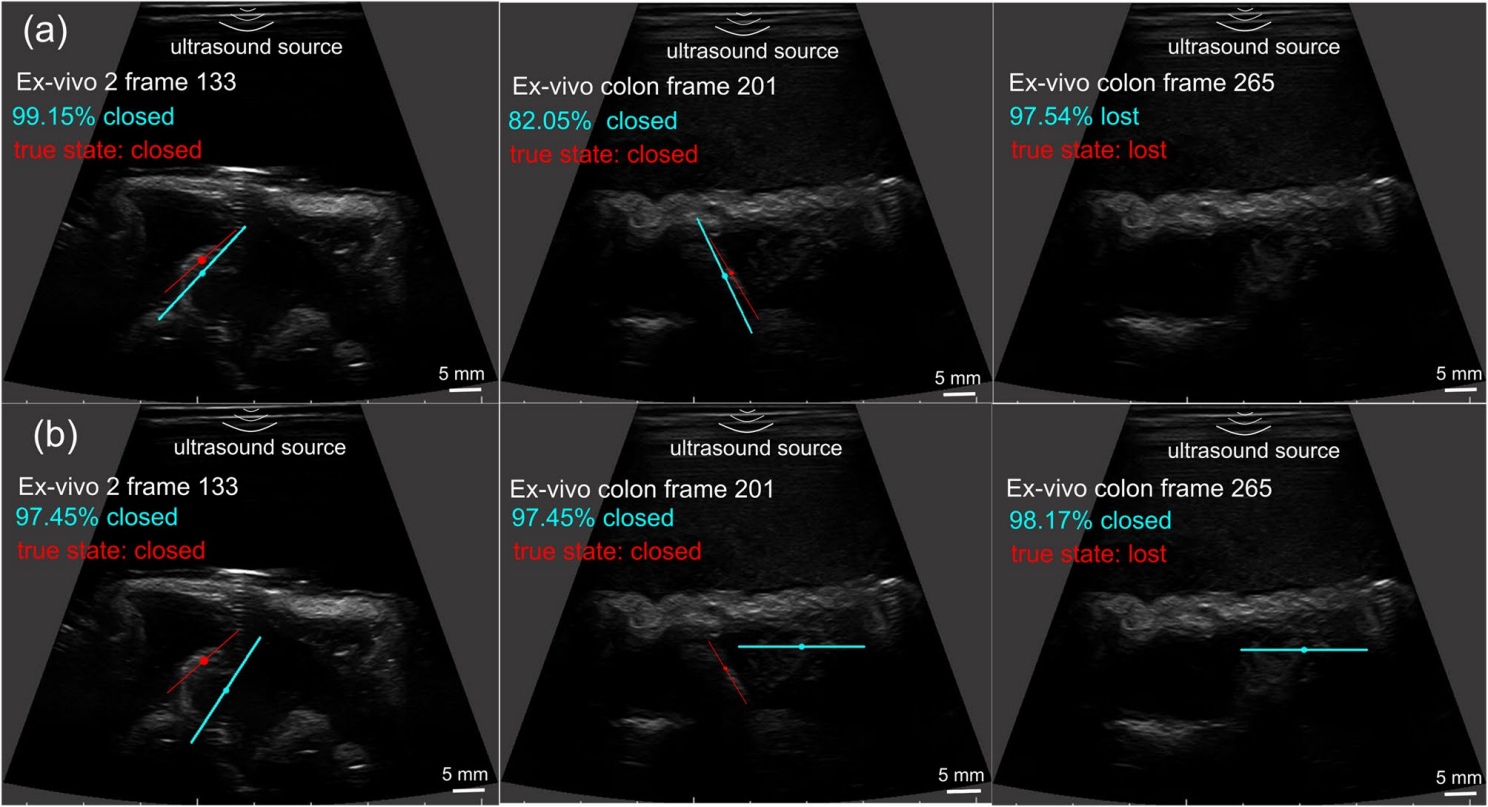
Comparison of the attention-based (**a**) and baseline model (**b**) for pose estimation and mechanism state detection of the capsule in three groups of frames from ex-vivo stomach and colon test datasets.

In addition to porcine stomachs, the trained neural network model was directly used to perform experiments in a porcine colon without further fine-tuning. The ex-vivo colon test images were generated using the same experimental setup, except that the porcine stomach was replaced with a section of colon tissue sample. Porcine colons feature large thickness of tissue layers, which renders low imaging contrast and occlusion of the capsule. Moreover, surrounding tissues and imaging artefacts bear high similarities with the capsule regarding the geometry and imaging contrast, which makes the accurate detection of the capsule more challenging. Despite of the difficult imaging conditions, our proposed method can still track the pose and status of the capsule successfully in this trial, especially in frames where the capsule presents a small region and low visibility due to occlusion, as shown in Fig. 5a. The model can also detect the “lost” status when the capsule is lost in the FOV, but has difficulty with recognizing closed capsule and provides the wrong state as “lost” in a few frames (Supplementary Fig. S4b). The complete tracking trajectory is shown in the supporting video.

### Ablation study

In this ablation study, we adopted the baseline model that has the same architecture with our proposed model but without the attention module to perform experiments on the hold-out test data, the ex-vivo porcine stomach and colon test sets to show the effectiveness of the attention module. The two neural network models were trained using the same training data and the two-stage transfer learning method. Supplementary Table 1 summarizes the quantitative results of the two methods on the hold-out and the first ex-vivo porcine stomach test data. Figure 5 compares the tracking results of the two methods for more challenging scenarios in three groups of frames where the images in the first row are identical to those in the second row, but with different tracking results provided by the proposed attention-based model and baseline model. Adding the attention module leads to noticeable performance gains under more difficult imaging conditions where the capsule presents a small region and vague contour definition with low visibility and noisier background, while the baseline model tends to be affected by the surrounding tissues and imaging artefacts. One explanation is that the attention module enhances the feature representation and allows the model focus on the target features while suppress background ones. Moreover, the attention-based model drastically improves the ability on detecting “lost” capsule and differentiating the closed capsule from tissues with similar features.

## Discussion and conclusion

This paper proposes an attention-based deep learning approach and adapts a pre-trained CNN with a natural image dataset to a clinical US dataset for simultaneous detection of the mechanism state and 2D pose of millimeter capsule robots in an ex-vivo pig’s GI tract using 2D B-mode US imaging. Since there are no existing benchmark datasets for our target use case, a clinically representative US dataset of the capsule robot is generated in ex-vivo pig’s stomachs to train the neural networks. Experimental results manifest that our proposed method achieves high detection accuracies on the unseen hold-out test data and has been successfully applied to detect the pose and mechanism state of the capsule in an ex-vivo porcine stomach and colon without further fine-tuning. Integrating the attention module with the feature extraction component improves the feature representation, which allows for the neural network model focus on target features in low signal-to-noise ratio environments, and thus significantly enhances the tracking performance in challenging cases where the capsule has low visibility and the presence of anatomical structures featuring predominant contrast and similar geometries with the capsule. Moreover, the trained model demonstrates good generalization ability to different anatomical areas in the GI tract and imaging settings without further fine-tuning, which shows great promise for transferring from ex-vivo to in-vivo environments.

However, failure cases occur in some frames, indicating the neural network model is not robust and capable of generalizing to all scenarios. This is due to the insufficient training data and it’s difficult to incorporate all possible imaging conditions, given that anatomical features vary in different porcine GI tract. Additionally, we design a clinically representative imaging setup by using an ex-vivo porcine stomach and the PEG solution dissolved with digestive enzymes to replicate the clinical GI ultrasonography. Nevertheless, there is still a gap between the designed imaging environment and in-vivo environment of both live animals and human in terms of acoustic properties of interior fluids and tissues, tissue contractions, especially the existence of air pockets. At the interface between surrounding soft tissues and gas pockets in the gut, most of the ultrasound waves are reflected and no further echoes can be obtained from beyond such an interface. Thus, the air pockets can result in a relatively low contrast and visibility of the capsule in the B-mode image if the capsule is located beyond the air gaps. In this case, the features such as the contour and geometry of the capsule are vague, which will be difficult for the deep learning model to learn and recognize and the model will fail to track the capsule. On the other hand, in-vivo artefacts are not directly learned by the model and may render the imaging contrast and contour definition of the capsule different from those in our dataset, which pose a challenge on the generalization capability of the neural networks model. In order to ensure the approach is reliable to be deployed in-vivo, the proposed model, as an ex-vivo pre-trained model, will be fine-tuned using in-vivo training data.

This is not in the scope for this paper. In our future work, we will address this issue more directly and modify our experimental setup to render a more realistic imaging environment by adding air pockets and obtaining the dataset for training the deep learning model, which will be more robust to the effect of the small air gaps in the gut and have a better generalizability to in-vivo imaging environments. Furthermore, real-time tracking will be achieved and integrated with an actuation system for the closed-loop control of the capsule robots with in-vivo applications on live animals.

## Methods

### Dataset generation

An agar/gelatin gel phantom has been widely used as an ultrasound phantom^37^. In the agar-gel phantom, the capsule appears with a higher contrast, and the edges of the capsule are much clearer than in the ex-vivo pig stomach (Fig. 1a). The automated tracking task is thus more challenging in the ex-vivo images; to enable clinical use it is necessary to develop and test such algorithms in real-tissue models. Obtaining large numbers of labeled in-vivo images of the capsule robot with accurate ground-truth location is logistically challenging. It’s appealing to train a deep neural network model using easy-to-obtain synthetic or simulated datasets as opposed to large-scale real-world data. But the discrepancy between simulated and real ultrasound images can be significant, which can affect the interpretability of images and the generalization ability of deep learning algorithms to real clinical scenarios. For these reasons and no existing benchmark datasets can be used for our target use case, an US dataset of the capsule robot in ex-vivo porcine stomachs was acquired and manually annotated with the capsule pose and status. We purchased the porcine stomach and colon samples from grocery, which has been processed and cleaned. No animal test or experiment were conducted in this work.

#### Image collection

Two base datasets were generated for the mechanism state classification and 2D pose estimation with a size of 2930 and 2631 images, respectively. US images were acquired using a clinical US system (Telemed ArtUs EXT-1H) with a linear array transducer (L15-7H40-A5), operating in wideview (trapezoidal) B-mode imaging mode. Before data collection, the imaging depth and frequency of the US system were set at 60-80 mm and 7.5 MHz, respectively, which are appropriate settings in clinical abdominal US imaging^38^. Other imaging parameters including the focus, power & gain, and dynamic range were manually adjusted each time to obtain satisfactory contrast. Five ex-vivo adult porcine stomachs with different sizes and tissue layer thicknesses were used during the data collection to create varying imaging environments.

During data collection for the 2D pose estimation, the probe was first placed at the center of the side wall of the container to image the cross-section of the full workspace. The initial capsule pose in ultrasound image coordinate system *U* is represented as $(x_0^{U}, y_0^{U}, \theta _0^{U})$. We take the center point of the scanned workspace plane as the origin and establish the global coordinate system *G* shown in Fig. 2b. Actuated by a permanent magnet at the top of the workspace 7 cm from the stomach surface, the capsule was rotated around the *z*-axis of *G* continuously from $0^{\circ }$ to $179^{\circ }$ to generate in-plane rotations, while the probe was translated from the center to the left and right along *x*-axis of *G* to generate images with more position variations, as shown in Fig. 2b. The assumption is that moving the scanner while keeping the capsule static in position is equivalent to driving the capsule to move in the workspace without out-of-plane movements. A total of 2631 images were acquired that span the entire possible positions of the workspace and full range of angles. The generated 2D pose of the capsule is denoted as $(x_i^{U}, y_i^{U}, \theta _{i}^{U})$, where $i\in \{ 1,2,\ldots ,N \}$ with *N* representing the total number of images in the base dataset. Then, the capsule was actuated by the magnet to open/close its sampling cavity with pose variations in the workspace to generate the dataset for the mechanism state classification, during which the probe was kept fixed to image from the side. We discarded the images where the capsule cannot be visualized as the open status since the open cavity could only be imaged within a specific range of viewing angles. The capsule was imaged at different depths within the workspace, and time gain control (TGC) was used to enhance the visibility of the capsule. Each image was first manually annotated using the four corner points that define the geometry of the capsule. The corner points were then converted to the center point $(x_{GTi}^{U}, y_{GTi}^{U})$ and orientation $\theta _{GTi}^{U}$ as the 2D pose labels of the capsule (Supplementary Fig. S1).

#### Data augmentation

Since we employ a supervised learning method, large numbers of annotated training images are needed to ensure the algorithm is robust to varying imaging conditions and generalize to unseen test data. However, our base dataset has too few images to train a robust deep neural network model. Thus, we employed data augmentation^39^ that is a common technique for increasing the amount of data in the machine learning domain, by applying known rotations and shifts to the acquired images in the base dataset, as shown in the Supplementary Materials.

The augmented dataset for the orientation was obtained by rotating each image in the base dataset around the image center by $5^{\circ }$ with a step of $0.5^{\circ }$ in a counter-clockwise direction, as shown in Fig. S2a,b in the Supplementary Materials. To be specific, from the original image *I* with dimension w$\times$h, the rotated image can be obtained by $I^{R}=I\times R$. The rotation matrix *R* is defined as $$\begin{bmatrix} \alpha &{} \beta &{} (1-\alpha )\cdot c_x - \beta \cdot c_y\\ -\beta &{} \alpha &{} \beta \cdot c_x + (1-\alpha )\cdot c_y \end{bmatrix}$$, where $\alpha =c\cdot \cos \theta$, $\beta =c\cdot \sin \theta$, with $\theta \in \ [0^{\circ },\, 5^{\circ }]$ being the rotation angle, $c_x$ and $c_y$ are the coordinates around which the image is rotated, *c* is a scale factor to resize the image after rotation. In our work, $c_x=\frac{w }{2}$ and $c_y=\frac{h }{2}$ are defined as the image center, and $c=1$. In order to generate a balanced dataset while not distorting the images, the data augmentation strategy was applied to complement the dataset with small missing angles and further expand the dataset. We also expanded the base dataset by shifting each image *I* using the defined translation matrix $$\begin{bmatrix} 1 &{} 0 &{} t_x\\ 0 &{} 1 &{} t_y \end{bmatrix}$$, where $t_x\in \ [-10,\, 10]$ and $t_y\in \ [-10,\, 12]$ are the pixels by which the image to be shifted along *x*- and *y*- axis while ensure the integrity of the capsule in the shifted images (Supplementary Fig. S2d). Black borders resulted from the rotation and shift were cropped to avoid the neural network using these black borders as features during the training process (Supplementary Fig. S2c,e). These new images (with accordingly shifted and rotated pose labels) and the base dataset images constitute an augmented dataset with a size of 52,620 images, which consists of 24230 and 28390 for 2D pose estimation and state detection, respectively. During training, we split the two training datasets into train, validation, and test set with a ratio of 7:2:1.

### Attention-based hierarchical neural network

**Figure 6:**
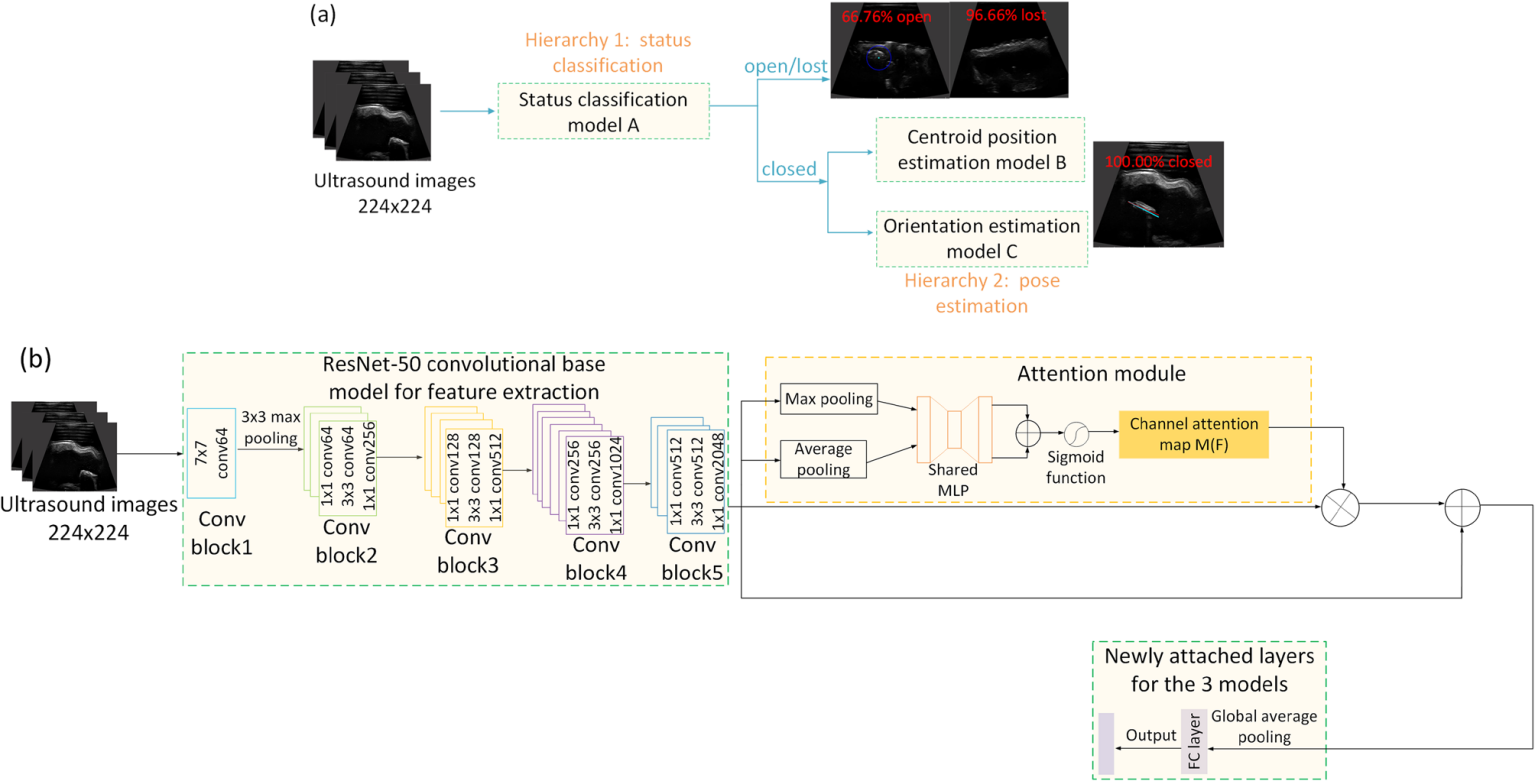
The hierarchical neural network. (**a**) depicts the hierarchical architecture and the workflow and (**b**) demonstrates the neural network architecture of the three models.

We proposed an attention-based hierarchical model to simultaneously detect the state and estimate the 2D pose of the capsule robot in US frames. The integration of the attention module can increase feature representation power, allowing the model focus on target features while suppress background noise. The workflow is shown in Fig. 6a, where Model A first performs state classification; if the detected state is closed, Model B and C for the 2D pose prediction are triggered and provide orientation and center point position estimations. Otherwise, the model detects the capsule as an “open” or “lost” state. Instead of training one model for multiple tasks, three models are trained separately for the mechanism state classification, centroid position, and orientation estimations using different training hyperparameters, including fine-tuning depths, learning rate and training epochs, to obtain optimal performances for different tasks.

The three models have a similar architecture, which consists of the pre-trained ResNet-50^40^ residual blocks for feature extraction, an attention module, a global average pooling (GAP) layer, and a fully-connected (FC) output layer with either a regression or classification output in the desired dimensions, as shown in Fig. 6b. We adopt ResNet because it solves the optimization difficulty of vanishing gradient for training deep neural networks and obtains faster convergence and accuracy gains from increased depth. Consider the *l*-th convolutional block in the pre-trained ResNet model:

3 $$\begin{aligned} F_l=f_l(F_{l-1})+F_{l-1} \end{aligned}$$

where $l\in \{ 1,2,\ldots ,L \}$ and $L=5$ is the total convolutional blocks in the ResNet-50. Then, the attention module is integrated with the ResNet output feature map $F_l\in R^{H\times W\times C}$ using the residual learning scheme to facilitate the gradient flow. We follow the channel attention concept proposed by Woo et al.^41^ where both a global max pooling layer (GMP) and global average pooling layer (GAP) are performed on $F_l$ to encode the most salient features and global statistics of average-pooled features from the feature map. After that, the two resultant tensors are forwarded to a shared two-layer multi-layer perceptron (MLP), and the output feature vectors are merged using element-wise summation. The attention map $F_l\in R^{1\times 1\times C}$ is computed as:

4 $$\begin{aligned} M(F_l)=\sigma (W_1(W_0(F_{GAP}))+W_1(W_0(F_{GMP}))) \end{aligned}$$

where $\sigma$ is the sigmoid function, $W_0$ and $W_1$ are the weights of the shared MLP, $F_{GAP}$ and $F_{GMP}$ denote the average-pooled features and max-pooled features, respectively. The refined feature map $F'\in R^{H\times W\times C}$ is then computed using the residual learning scheme as $F'=F_l\oplus (F_l\otimes M (F_l))$, where $\oplus$ and $\otimes$ represent element-wise summation and element-wise multiplication, respectively.

We formulate the orientation estimation problem using a classification framework which trains deep neural networks to classify orientations into a discretized pose space (180 classes corresponding to $0^{\circ }\sim 179^{\circ }$). In this way, the convergence issues introduced by the representational constraints and pose ambiguities when using a direct regression model can be avoided, and classification methods investigated in the literature have generally shown to work better than direct regression approaches for object orientation estimation^42^. The output for the state classification and orientation estimation model is configured with the Softmax output layer with 3 and 180 classes, respectively. The center-point position estimation is formulated as a deep regression problem, in which the output layer has a linear function. In the three models, Dropout is used after the GAP layer to avoid overfitting and the Rectified Linear Unit (ReLU) function is used for all internal nodes of the neural network.

### Transfer learning for the mechanism state detection and 2D pose estimation

In this work, we adapt the success of transfer learning^43^ in computer vision towards solving a multi-task tracking problem in ex-vivo tissue environments with limited ultrasound dataset. However, the ResNet model is pre-trained on a natural image dataset like ImageNet for object classification, which is fundamentally different from our ultrasound dataset and the target tracking tasks. Transferring the pre-trained model with large domain gap for a different target application is challenging. The features generated from the convolutional base gradually transform from more general and reusable features at lower layers to more abstract features at higher layers, which results in a large transferability gap when transferring pre-trained features to a dissimilar task and degrades the model performance. Thus, we employ the two-stage transfer learning method (Supplementary Fig. S3) where the ResNet base model is initialized with the pre-trained weights and then the model is fine-tuned with an appropriate fine-tuning depth (FT)^44^, defined as the deepest block fixed during fine-tuning, to improve the model convergence and generalization.

### Model training

#### Data preprocessing

Before training the model, the images were normalized and resized to 224$\times$224 pixels to fit the required input dimensions of ResNet. Accordingly, the 2D pose labels were updated using the following equations: $\theta '= \frac{w }{h }tan\theta$, $x'=\frac{224x}{w }$, $y'= \frac{224y}{h }$, where $\theta$ and $\theta '$ are the original and updated orientation labels after resizing, respectively. Dimensions $w$ and $h$ denote the width and height of the original image. $(x,y)$ and $(x',y')$ represent the centroid position coordinates in the original and resized images, respectively.

#### Training

The network models were implemented in TensorFlow and trained for 11 hours in the Win10 Anaconda virtual environment with TensorFlow GPU (using an NVIDIA GeForce RTX 2070). All the layers of the convolutional base model were initialized with pre-trained ImageNet parameters, while the layers in the attention module and the output FC layers were initialized with random parameters. Then the proper feature map $F_{l}$ output from the ResNet base model was chosen as the input to the attention module. The training process contains two stages. First, the ResNet convolutional base model was frozen, and only the randomly initialized layers were trained using Adam optimizer and a learning rate (LR) of $10^{-4}$. Then, the base model was unfrozen, and the model was fine-tuned using a low LR of $10^{-5}$ with an FT of $CB^{i}$, where $CB^{i}$ represents the (*i*th) convolutional block of ResNet-50 fixed during training. Our ultrasound dataset greatly differs from the pre-trained ImageNet data, fine-tuning higher layers to adapt the pre-trained model to our new dataset is critical to improving the model performance. The models were trained on the datasets that were split into training, validation, and test sets with a ratio of $7:2:1$ using a batch size of 32.

The status classification model was trained on the dataset generated by augmenting the status base dataset through shift and rotation. The augmented dataset contains 28,388 images with 9810, 9688, and 8890 frames labeled as open, closed, and lost capsules. We used $F_{l}=4$ and the model was fined-tuned using an FT of $CB^2$ and the cross-entropy cost function

5 $$\begin{aligned} L=-\frac{1}{N}\sum _{i=1}^{N}\left(\sum _{k=1}^{K}{{\textbf {t}}}_k^{(i)}\log {{\textbf {y}}}_k^{(i)}\right) \end{aligned}$$

where $K$ denotes the classification labels of 3 classes, ${{\textbf {t}}}_k^{(i)}$ and ${{\textbf {y}}}_k^{(i)}$ are the ground truth and predicted probability for each of the class, *N* is the total number of training images. The model converged after training for 65 epochs, and overfitting is prevented by using early stopping, dropout with a ratio of 0.5. The 2D position regression model which used $F_{l}=5$ was trained on 25, 210 images to regress the centroid position of the capsule, and converged after training for 102 epochs. The model was fined-tuned using an FT of $CB^2$ and trained using the mean squared error (MSE).

6 $$\begin{aligned} L=\frac{1}{2N}\sum _{i=1}^{N}\Vert {{\textbf {y}}}_p^{(i)}-{{\textbf {t}}}_p^{(i)}\Vert \end{aligned}$$

where ${{\textbf {t}}}_p^{(i)}$ and ${{\textbf {y}}}_p^{(i)}$ are the ground truth and predicted center point coordinates of the capsule, respectively. In addition, we deployed a classification model with 180 classes corresponding to $0^{\circ }\sim 179^{\circ }$ to estimate the orientation of the capsule. The feature map *F* was the output of the (5th) convolutional block. The entire model was fine-tuned and trained using the cross-entropy loss in equation (3). After training for 60 epochs, the cost function converged to a global minimum value.

## Supplementary Information

Supplementary Information 1.

Supplementary Information 2.

## Data Availability

The dataset generated during the current study are available in the following repository at https://drive.google.com/drive/folders/1aLTuf9D7L8gdYWOuEiET_GQ0uWIrJww-?usp=sharing
