# Supplementary material for: Capsule robot pose and mechanism state detection in ultrasound using attention-based hierarchical deep learning
Source: Sci Rep. 2022 Dec 7;12:21130. doi: 10.1038/s41598-022-25572-w (PMC9729303; doi:10.1038/s41598-022-25572-w)
Supplement: Supplementary file 1 — Supplementary Information 1. [file 41598_2022_25572_MOESM1_ESM.pdf]

Capsule Robot Pose and Mechanism State Detection in Ultrasound using Attention-based Hierarchical Deep Learning

Xiaoyun Liu[1], Daniel Esser[2], Brandon Wagstaff[3], Anna Zavodni[4], Naomi Matsuura[5], Jonathan Kelly[3], and Eric Diller[1,*]

[1]Department of Mechanical and Industrial Engineering, University of Toronto, Toronto, ON M5S1A8, Canada
[2]Department of Mechanical Engineering, Vanderbilt University, Nashville, TN 37235, USA
[3]University of Toronto Institute of Aerospace Studies, University of Toronto, Toronto, ON M5S1A8, Canada
[4]Department of Medicine, Division of Cardiology, University of Toronto, Toronto, ON M5S1A8, Canada
[5]Department of Materials Science & Engineering and Institute of Biomedical Engineering, University of Toronto, Toronto, ON M5S1A8, Canada
[*]ediller@mie.utoronto.ca

Supplementary Information

2D Pose Labels Generation

Each image is first manually annotated using the four corner points that defines the geometry of the capsule. Then a binary mask defined by the corner points is generated for each image using OpenCV. Contour detection is then applied on the binary mask where all the noise and artefacts are removed to obtain the contour center point pixel coordinate (x, y) and orientation θ as the 2D pose labels of the capsule as shown in Figure S1.

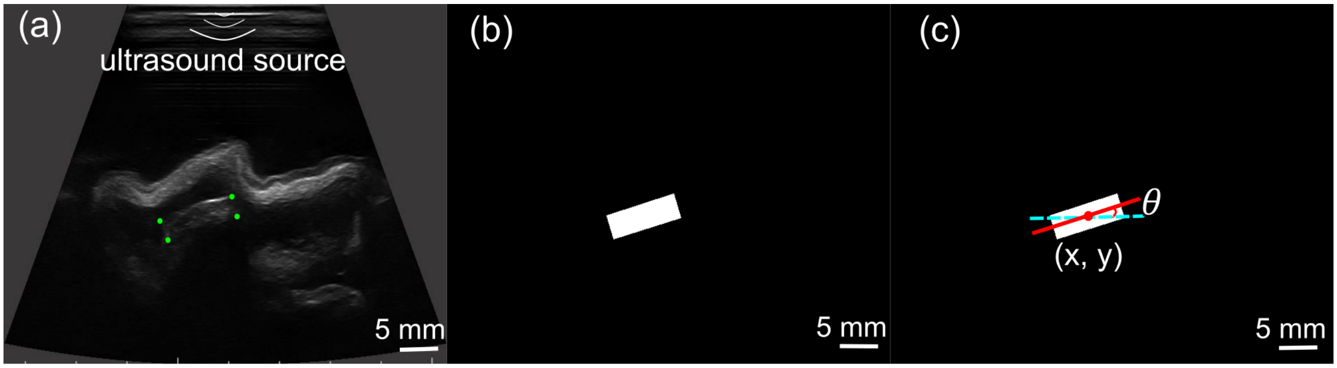

Figure S1. 2D pose labels generation including the raw ultrasound image with the annotated corner points (a), generated binary mask defining the capsule's geometry (b) and 2D pose label including centroid position and orientation of the capsule (c).

Data Augmentation

We employed data augmentation by applying known rotations and shifts to the acquired images in the base dataset to enlarge the training dataset.

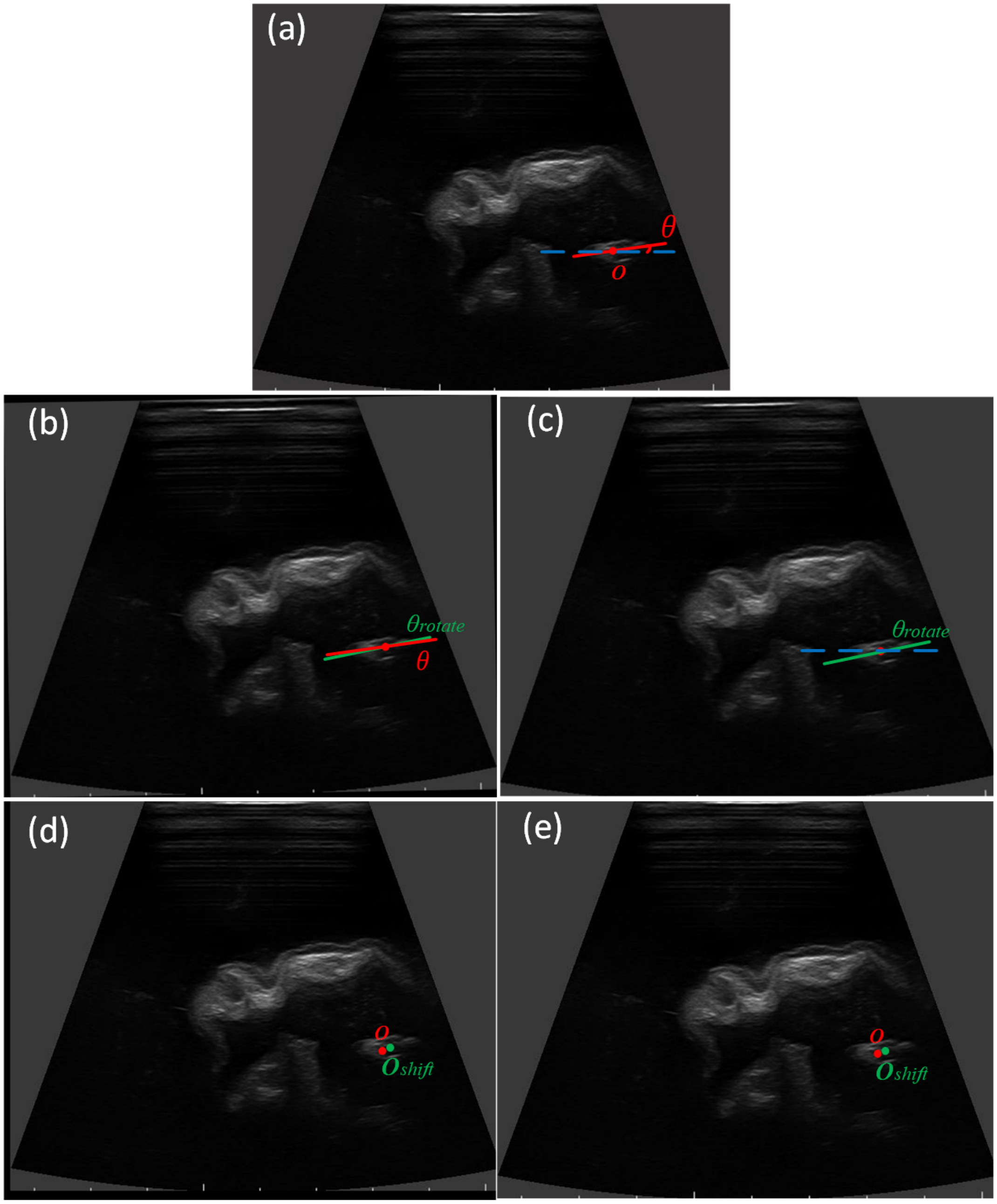

Figure S2. Data augmentation method. (a) is the original image from the base dataset. (b) and (c) show the rotated image and the image without black borders after rotation. (d) and (e) are the translated image and image without black borders after translation.

Two-stage Transfer Learning

We employed the two-stage transfer learning method where the ResNet base model was initialized with the pre-trained weights and then the model was fine-tuned with an appropriate fine-tuning depth (FT), defined as the deepest block fixed during fine-tuning, to improve the model convergence and generalization.

Failure Detection Cases in Ex-vivo Porcine GI Tract

When the capsule gradually opens or the tissue occludes the cavity, the neural network model has difficulty with recognizing the open state and erroneously detects the closed state. The model also has difficulty with recognizing closed capsule and

provides the wrong state as “lost” in a few frames in the porcine colon.

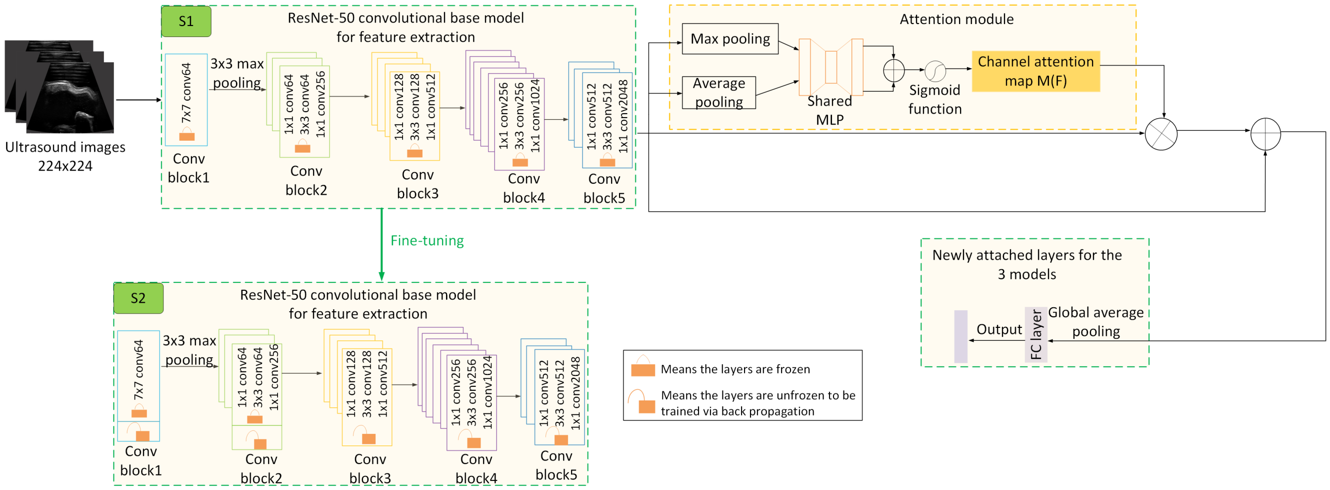

Figure S3. Schematic for the training process, where S1 and S2 denotes the two training stages. The status detection model uses the output of the 4th Conv block as the feature map, while the other two models use the output of 5th Conv block as the feature map. In S1, all the layers of the base model are frozen. In S2, all the layers are fine-tuned for the orientation model, represented by the “unlock” symbol, while only the layers after the Conv block2 are fine-tuned for the centroid position model and status detection model.

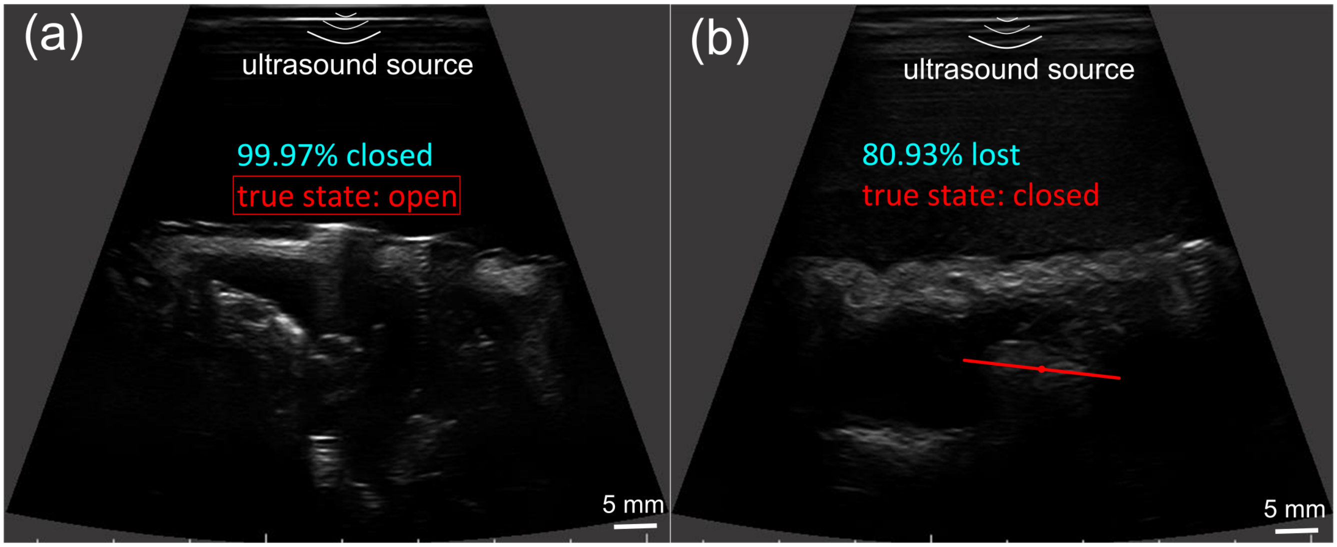

Figure S4. Snapshots from the ex-vivo tracking of the capsule in the porcine stomach and colon showing the failure tracking frames where the capsule is erroneously detected as closed state (a) and lost state (b).

Ablation Study on Hold-out and the First Ex-vivo Test Datasets

Table S1 summarizes the quantitative results of the two methods on the hold-out and the first ex-vivo porcine stomach test data. The attention-based model achieves similar detection accuracy with the baseline model on these two test sets, although the baseline model has better performance on orientation estimation on the hold-out test set. However, the attention-based orientation model outperforms the baseline counterpart on the ex-vivo test set with higher accuracy, demonstrating better generalization capability to different imaging environments.

Ablation study on the hold-out test set			
Architecture	Status classification accuracy	Centroid position mean error (mm)	Orientation mean error (degrees) with successful rate
Attention-based model	97.83%	0.24	2.0, 97.8%
Baseline model	95.03%	0.29	1.6, 99.3%
Ablation study on the first ex-vivo test set			
Attention-based model	100%	1.75	3.5, 96.9%
Baseline model	100%	1.31	4.1, 92.5%

Table S1. Ablation study on the hold-out and first ex-vivo test set.
